# The Role of Succinic Acid Metabolism in Ovarian Cancer

**DOI:** 10.3389/fonc.2021.769196

**Published:** 2021-11-02

**Authors:** Lei Xia, Hairong Zhang, Xuezhen Wang, Xiaoyu Zhang, Ke Nie

**Affiliations:** ^1^ Department of Pathology, Shandong University of Traditional Chinese Medicine, Jinan, China; ^2^ Department of Obstetrics and Gynecology, Shandong Provincial Third Hospital, Jinan, China; ^3^ School of Chinese Medicine, Shandong University of Traditional Chinese Medicine, Jinan, China; ^4^ School of Chinese Materia Medica, Guangdong Pharmaceutical University, Guangzhou, China

**Keywords:** succinate acid, glutamine, IDH, SDH, SUCNR1

## Abstract

Ovarian cancer is one of the most common malignancies and the highest mortality among gynecological malignancy. The standard therapy options for patients with ovarian cancer are cytoreductive surgery and chemotherapy, and although most patients do better with standard treatment, it is easy to relapse and be resistant to chemotherapy. Therefore, it is important to find new therapeutic strategies. More recently, metabolic reprogramming has been recognized as a hallmark of cancer and has become a potential target for tumor therapy. Mutations of metabolic enzymes are closely related to the development of ovarian cancer. The metabolic reprogramming of ovarian cancer not only provides energy to tumor cells, but also participates in various biological processes as signaling molecules. Succinic acid (SA) is an important metabolic intermediate involved in a number of metabolic pathways, such as TCA cycle and glutamine metabolism, and is also widely present in a variety of plants and vegetables. Studies show abnormal SA metabolism in many tumors and affect tumor formation through a variety of mechanisms. But the role of SA in ovarian cancer is less studied. This paper reviews the role of SA and its abnormal metabolic pathway in ovarian cancer.

## Introduction

Ovarian cancer is one of the most common gynecological malignancies, and is the highest mortality of gynecological malignancies (1). Most patients are advanced so the prognosis is poor (2). The standard treatment is cytoreductive surgery and platinum/taxane combination chemotherapy. The response rate to first-line therapy is around 80%. but most patient relapse and develop chemotherapy resistance (3–5). Therefore, it is urgent to find new treatment strategies clinically.

The microenvironment of ovarian cancer cell is characterized by ascites, hypoxia and low level of glucose (6, 7). Therefore, metabolic reprogramming is an important characteristics of ovarian cancer cells (8). Metabolic reprogramming can not only help in tumor cell survival and proliferation, but also affect tumor cell migration and the chemotherapy resistance formation (9). A good understanding of ovarian cancer cell metabolism reprogramming contributes to a better understanding of the occurrence mechanism of ovarian cancer, and also helps in finding new treatment measurements (10). This metabolic reprogramming can not only guarantee the energy source of tumor cells, but the metabolic intermediates can also be involved in tumor formation as signal molecule (11).

## The Role of SA in Metabolism

SA was discovered in 1546, and it was one important intermediates involved in a variety of metabolic pathways. It is now thought to be closely related to tumorigenesis (12). In TCA cycle, isocitric acid is transformed into α-ketoglutaric acid (α-KG) under the action of isocitrate dehydrogenase (IDH). α-ketoglutarate dehydrogenase (α-KGDH) catalyses α-KG to form succinyl-CoA. Succinyl-CoA succinatethiokinase then catalyses the hydrolysis of succinyl-CoA to form SA. Succinate dehydrogenase(SDH) catalyses the oxidation of SA to fumarate. Fumarate dehydrogenase(FH) catalyses the hydration of the α,β-unsaturated carbonyl compound fumarate to form malic acid. IDH and α-KGDH are the rate-limiting enzymes in the TCA cycle. When increasing of the IDH activity, or decreasing of SDH or FH activity can leads to the accumulation of SA in the tumor cells (13, 14).

Metabolic intermediates in TCA cycle, such as α-KG, can also participate in other metabolic processes, thereby causing changes of SA concentration in tumor cell (15). For example, α-KG can form glutamate when acting by transaminase, participating in the glutamine cycle, while leading to TCA cycle process is weakened (16). Glutamine decomposition produces a α-KG (complement reaction) increases the TCA cycle process and gives cells more energy (17). The complement reaction involves two enzymes, glutamase (GLS), and glutamate dehydrogenase (GDH), in which GLS is the speed-limiting enzyme for the process. Glutamine can also further generate other substances such as GABA under the action of glutamate decarboxylase.

SA can also be generated from other precursors, including Υ-aminobutyric acid(GABA) and glyoxylate (18). Abnormal function of multiple metabolic pathways and related enzymes can lead to abnormal accumulation of SA in tumor cells.

## Abnormal Succinic Acid Metabolism in Ovarian Cancer

### IDH Mutation and Ovarian Cancer

IDH inactivation causes blocked TCA circulation, resulting in energy generation disorders and the decrease of a-KG and SA (12). However, the role of IDH mutation in tumorigenesis is known with the production of tumor metabolites D-2HG (19, 20). D-2HG is an analogue of α-KG that inhibits the a-KG-dependent dioxygenase. The accumulation of D-2HG in ovarian cancer has been found, but studies showed that IDH abnormalities in ovarian cancer are mainly manifested by wild-type IDH overexpression (21). It was found that IDH expression was significantly increased in OVAR 3 and OVARIAN 10 cells compared to normal epithelial cells. IDH expression has a very important role in maintaining TCA to reduce aerobic glycolysis processes and promote ATP generation. When IDH inhibitors or genetic silencing of IDH are used, tumor cells have significant aging phenomena while aging-related markers such as beta galactosidase,PML bodies and lamin B1 significantly decreased (22). It is known that IDH is inhibited leads to reduced NADH, resulting in a significant increase in ROS, which inducing cell aging. But studies have shown that the relationship between IDH inhibition and aging is independent of ROS (8).

### SDH Mutation and Ovarian Cancer

SDH, as a tumor suppressor, gene consisting of six subunit encoding of SDHA, SDHB, SDHC, SDHD, SDHAF1, SDHAF2 (23). Genetics mutations of SDH have been found in some types of cancer such as paraganglias or kidney cell cancer, and SDH downregulation has been observed in gastric and colon cancers. Data analysis shows, there is a high probability of SDH amplification in high-grade ovarian cancer. Amongst which amplification of SDHA reach 14%, and amplification of SDHB、SDHC、SDHD、SDHAF1、SDHAF2、SDHAF3 is 2%、5%、4%、9%、1% and 2% respectively. There are significant differences between different cells, such as OVCAR3 with SDHAF1 amplification, which means that SDHA expression is enhanced. A2780 cells have relatively low expression based on SDHA compared to OVCAR3 (24).

SDHB silencing promoted cell proliferation, invasion, and migration, but inhibited apoptosis of SKOV3 and A2780 cells. In contrast, overexpression of SDHB inhibited cell proliferation, invasion, migration, and promoted apoptosis in SKOV3 cells (25). It was observed that upregulation of Bcl-2 and MMP-2, activation of p-P38, p-ERK, and p-FAK, inhibition of cleaved caspase 3 in SDHB-silenced cells. HIF-1α, an essential factor in tumor progression, was upregulated in SDHB-silenced cells with the activation of p-AMPKα and down-regulated in SDHB-overexpressed cancer cells with the decreased p-AMPKα. And SDHB was proved to be decreased due to upregulation of HIF-1α expression in CoCl2-treated cancer cells.

### SDHAP1 and Ovarian Cancer

Succinate dehydrogenase complex flavoprotein subunit A pseudogenene 1(SDHAP1) is located on chromosome 3, encoding lncRNA SDHAP1 which is associated with chemoresistance in ovarian cancer. Studies have been shown that SDHAP1 was upregulated in PTX-resistant SKOV3 and Hey-8 ovarian cancer cell lines, while miR-4465 levels were down-regulated. Silencing of SDHAP1 induced re-acquirement of chemo-sensitivity to PTX in ovarian cancer cells *in vitro*. In mechanism, SDHAP1 upregulates EIF4G2 expression through sponge miR-4465, thereby promoting PTX induced apoptosis in ovarian cancer cells. The regulatory networks involving SDHAP1, miR-4465 and EIF4G2 participate may be potential therapeutic targets for PTX-resistant ovarian cancer (26).

### Glutamine Metabolism in Ovarian Cancer

The growth of some tumor cells is depended on glutamine, a phenomenon referred to as “glutamine addiction”. Glutamine has been reported as essential for the proliferation and metastasis of OVCA cells (27).

C13K and SKOV3 cells were treated in 48 hours of glutamine-free media with various concentrations of glutamine (0, 2.0, 5.0, and 10.0 mM), and found that it directly maintained the proliferation of cancer cells. The studies believe that normal cells can use glucose and glutamine for energy, but that tumor cells can obtain energy through glutamine during glucose utilization disorders. When using mTOR inhibitors, glucose intake of SKOV3 and C13K cells decreases and glucose metabolism is significantly inhibited, but glutamine metabolism is not enough to block, so that tumor cells can still produce sufficient energy to sustain cell proliferation. When glucose and glutamine metabolism were inhibited, mTOR inhibitor induced apoptosis of ovarian cancer cells was more obvious (28).

Similar experiment is also confirmed in the HEY and IGROV-1 ovarian cancer cell lines (29). Moreover, the glutamine inhibit GLS expression with a dose-dependent manner, with mechanisms that may be related to regulating the MAPK and mTOR/S6 pathways. Further studies show that glutamine can reduce p21 expression by increasing cyclin D1, CDK4, thus pushing ovarian cancer cells from stage G1 to stage S. ROS levels and the expression of PERK, PARP, Calnexin and Bip increased significantly after glutamine deprivation. After increasing the glutamine supply, the ROS levels were significantly reduced, and the relevant indicators also declined. It shows that glutamine can regulate cellular oxidative stress and endoplasmic reticulum stress. The authors argue that targeted glutamine metabolism may be a promising therapeutic strategy in the treatment of ovarian cancer.

GLS is a speed-limiting enzyme that transforms from glutamine to glutamate. There are two genes that code for glutaminase in the human genome. Chromosome 2 codes for glutaminase 1 (GLS1) and glutaminase 2 (GLS2) is located on chromosome 12. In patients with epithelial ovarian cancer, the levels of GLS1 is negatively correlated with prognosis. Further studies have shown that GLS1 has many isoforms, such as the GAC isoform (genomic exon 1-15) and the KGA isoform (genomic exons 1-14 and exons 16-19 with intact 3’-UTR). The GAC form has no known miRNA binding sites, whereas the KGA isoform contain a well-known miR23 binding site making them subject to miRNA regulation (30).

The study has shown that glutamine-dependent SKOV3 ovarian cancer cells express higher levels of the GAC and KGA isoforms than the glutamine independent and immortalized human fallopian tube secretory epithelial cells (hFTSECs). Dual knockdown of both by RNAi or inhibition by BPTES sensitized ovarian cancer cells to chemotherapy, regardless of their dependence on exogenous glutamine (30).

Conversely, glutamate is metabolized into glutamine by glutamine synthetase (GS). The level and function of GS in tumors vary depending on the cell background. Low invasive ovarian cancer cells express high levels of GS, while high aggressive ovarian cancer cells express low levels of GS. At the same time, GS promotes the biosynthesis of nucleotides and the growth of various cancer cells (27).

### ALDH5A1 Mutation and Ovarian Cancer

Aldehyde dehydrogenase ALDH5A1 encodes succate hemialdehyde dehydrogenase (SSADH), an enzyme involved in intracellular glutamate metabolism, associated with the synthesis of retinoic acid and GABA (31). One clinical study shows that ALDH5A1 single-nucleotide polymorphisms were significantly associated with ovarian cancer. Data analysis showed that ALDH5A1 expression was significantly reduced compared in ovarian cancer tissue to normal ovarian tissue. Moreover, ALDH5A1 expression was negatively associated with the prognosis of ovarian epithelial cancer. Upregulation in ALDH5A1 expression in patients with P53 mutants, but no significant relationship with prognosis in wild-type p53 (31, 32).

## SA Can Strengthen the TCA Cycle in Ovarian Cancer

**Figure 1:**
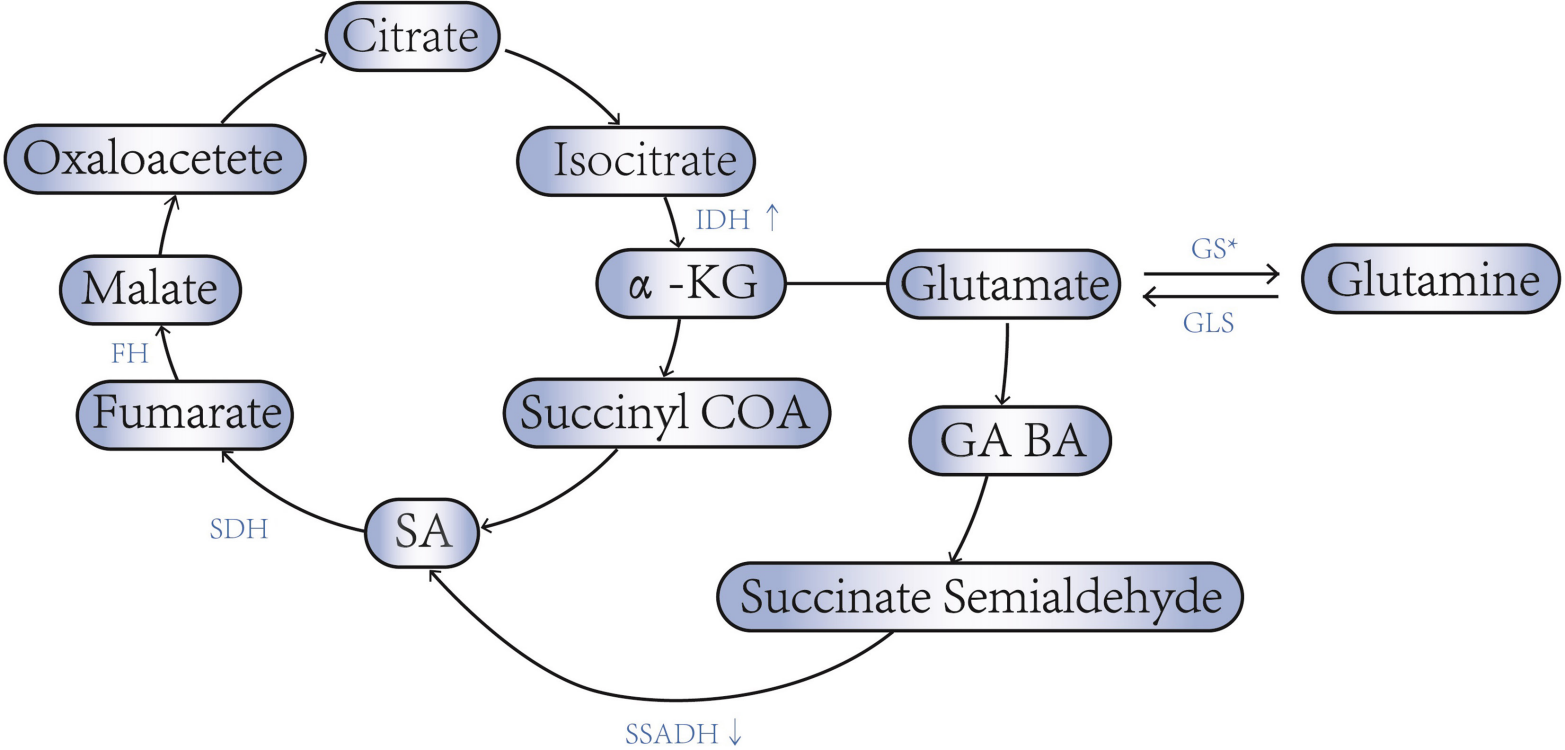
SA - associated enzyme abnormalities in ovarian cancer. * Low invasive ovarian cancer cells express high levels of GS, while high aggressive ovarian cancer cells express low levels of GS.

An important feature of metabolic reprogramming in tumor cells is aerobic glycolysis (Warburg effect), as a result of which metabolic intermediates may be involved in tumor proliferation, invasion, and metastasis (33). However, in ovarian cancer, aerobic glycolysis is not obvious, and its main feature is that the production of ATP is maintained by aerobic oxidation through TCA under the condition of hypoxia (34). As mentioned above, the activities of IDH, KGDH, SDH, GLS and other related enzymes are often enhanced, while the activities of SSDH and GS are decreased in ovarian cancer (**Figure 1**). This maintains TCA by increasing intermediates of TCA. Thus, the accumulating of SA in ovarian cancer may promote energy production by maintaining TCA From maintaining the TCA cycle, increased SA will promote cell energy generation and contribute to cell proliferation and migration.

Studies have also shown an accumulation of SA in patients with ovarian cancer. Ting Jiang (35) detected SA concentration in urine using the liquid chromatography four-link technology and found significantly increased urine SA concentrations in patients with ovarian cancer compared with normal controls. And its content increases with the development of the disease. Urinary SA was associated with clinical stages in patients with ovarian cancer. From the cellular level, studies have aslo shown that the metabolites associated with the TCA circulation, including SA, generally increase in high-grade ovarian cancer (36).

## The Role of SA in Ovarian Cancer

### SA and SUCNR1-Mediated Signaling Pathway in Ovarian Cancer

SUCNR1, also known as GPR91, is a member of the G protein coupled receptor family. SUCNR1 has been recognized as an orphan receptor until it was found to be able to bind to SA. The role of SUCNR1 has been fully demonstrated in renin induced hypertension, ischemia/reperfusion injury, inflammation and immune response, platelet aggregation and retinal angiogenesis (37). SUCNR1 is mainly expressed in kidney, heart, liver, retina, small intestine and other organs. Bioinformatics studies (38) showed the expression of SUCNR1 in serous epithelial ovarian cancer was significantly higher than in normal ovarian tissue, and there was no significant difference in SUCNR1 expression between serous and endometrial ovarian cancer cells, but the expression of SUCNR1 in clear cell carcinoma was significantly lower than that in serous ovarian cancer. But the relationship between the intracellular signal pathway of SUCNR1 and its pathophysiological role is not clear.

SUCNR1 expression was significantly increased in MUC16/CA125 mutant cells, but the mutations in TP53, PTEN, KRAS, BRCA1/2 and PI3K3CA had no significant effect on SUCNR1 expression.

The progression-free survival (PFS) was significantly shortened in patient with high expression of SUCNR1, but the overall survial (OS) was not significantly abnormal.

### SA With α-KG-Dependent Dioxygenase in Ovarian Cancer

Succinate can regulate the activity of the a-KG-dependent dioxygenase family members (12,39). For example, the ten eleven translocation (TET) and Jumonji-C-domain-containing histone demethylases (JMJDs). The TET protein can catalyzes the conversion of 5-methylcytosine to 5-hydroxymethylcytosine, and it is an important enzyme in DNA demethylation and the JMJDs is the largest family of histone demethylase. These enzymes are subject to product inhibition by succinate, and therefore, their activity is dependent on the ratio of α-KG to succinate. In this way, succinic acid can cause epigenetic changes in ovarian cancer cells. Epigenetic changes can alter how genes function, acting as switches. Importantly, changes in the expression of these genes are heritable and appear to be closely related to the metabolic state of the cell. Succinate may also indirectly regulate the activity of histone demethylases through their effects on HIF-1α, which can bind to and induce the expression of certain histone demethylases (40).

### Succinic Acid With HIFs in Ovarian Cancer

Under aerobic conditions, HIFs is hydroxylated at 564 and 402 under the action of proline hydroxylase, which binds to ubiquitin proteasome, resulting in HIFs degradation. In addition, HIFs inactivation occurs when dioxygenase hypoxia-inducible factor inhibitors hydroxylate at HIF803 in an oxygen-dependent manner. These reactions were inhibited under hypoxia. HIFs can bind to anoxic response elements and induce the expression of related genes. Genes known to be regulated by HIFs are involved in: ① erythropoiesis and iron metabolism; ② Angiogenesis, such as VEGF, COX2, etc.; ③ Cell proliferation, such as IGF-2; ④ Apoptosis, such as bcl-2, p53, p21, etc.; ⑤ Glucose metabolism, etc. Therefore, it plays an important role in tumorigenesis (41, 42).

The mechanism of succinic acid inducing HIFs mainly involves two aspects. One is that succinic acid, as an antagonist, can inhibit the activity of α-Kg-dependent dioxygenase, thus inhibiting the activation of related enzymes, so as to maintain the non-degradation of HIFs (15, 43). Succinic acid, on the other hand, can stabilize HIFs through The PI3K signaling pathway through SUCNR1 receptor (13, 37).

At present, there is no direct evidence to prove the effect of succinic acid intervention on HIFs, but some articles have suggested that changes in metabolic pathways can interfere with the production of HIFs.

SiRNA was used to silent and overexpress SDHB in SKOV3 and A2780 cells. It was found that when the function of SDHB was lost, the content of succinic acid was increased, and the proliferation, invasion and metastasis of tumor cells were enhanced, but the apoptosis process was inhibited. On the contrary, the proliferation, invasion and migration of cells were decreased due to the overexpression of SDHB. Apoptosis was also induced. Inhibition of SDHB can promote the up-regulation of HIFs expression by activating P-AMPK (25).

### SA and Tumor Immunity in Ovarian Cancer

Macrophages are an important cell component in the tumor microenvironment, and can activate and form type M2 tumor-related macrophages (TAM) under various signal regulation, which promotes the evolution of the tumor, and suppresses the anti-tumor immune response (44, 45). In several studies it was found that SA can induce conversion of tumor-associated macrophages (TAM), a process also associated with the SUCNR1 receptor, and that SA-induced TAM conversion was inhibited after anti-SUCNR1 antibody treatment (46, 47). Data analysis showed a significant positive correlation between tumor-related macrophage M2 cell marker expression and SUNCR1 expression in ovarian cancer. It is therefore believed that succinate acid can induce TAM through the SUCNR1 receptor.

The high expression of SUNCR1 causes different subsets of immune cells infiltration in ovarian cancer (48). For example, the expression of SUNCR1 was significantly positively correlated with the expression of CD8+ T cells, neutrophils, NK cells, TAM and M2 cells. Meanwhile, the SUNCR1 expression was positively correlated with the expression of regulatory T cells (such as Th1, and Th2). (GSE9891). It means that SA can be widely involved in immune regulation of ovarian cancer through SUCNR1, with mechanisms associated with multiple biological processes such as T cell activation, interleukin signaling, chemokine signaling, antigen processing and delivery, natural killer cell-mediated cytotoxicity, PD-1-blocked cancer immunotherapy, adaptive immune system and interferon α/β signaling, etc. The prognosis of ovarian cancer patients with high expression of SUCNR and large infiltration of neutrophils is poor. But the prognosis was better with high expression of SUCNR and large infiltration of M1 cells (38).

### SA and Oxidative Stress in Ovarian Cancer

Data analysis shows that ovarian cancer cells can be divided into two subtypes: high phosphorylation and low phosphorylation, based on the oxidative phosphorylation levels (49). Both can generate energy through glycolysis, but highly phosphorylated ovarian cancer can use glutamine and fatty acids to participate in the TCA cycle. Although there was no difference in Ki index and mitosis between the two cell types, oxidative stress was significantly increased in the highly phosphorylated type. At the same time, ROS and lipid peroxides were significantly increased, and the level of iron in intracellular lysozyme was significantly increased. High phosphorylated tumor cells are better sensitive to chemotherapy, which the authors suggest may be associated with ROS and iron death. However, studies have shown that cisplatin-resistant A2780 has shown significantly increased levels of glutamine, glutamate, and glutathione in cells compared to cisplatin-sensitive A2780 cells. Cell resistance to cisplatin was significantly reduced after glutamine deprivation, as did glutathione levels. It indicates that the formation of chemotherapy resistance is related to the generation of ROS (8).

### Succinic Acid and the Cell Cycle

Glucose metabolism can influence the cell cycle of ovarian cancer. When the activation of G6PC was enhanced, it not only increased cellular glucose uptake, but also inhibited CDKN1B, which is inhibiting CDK2. This suggests that glucose metabolism is closely related to the cell cycle, especially the CDK2/cyclin E axis. CDK inhibitor alone performed poorly on cell cycle inhibition of ovarian cancer cells. The TCA metabolism of A2780 cells was significantly enhanced by CDKi. but the changes in oxyglycolysis and pentose phosphate pathway in tumor cells were insignificant. Therefore, it is believed that ovarian cancer cells treated with CDKi can increase energy production through the TCA pathway to combat the cell cycle arrest caused by CDK inhibitors. Lower SDHA gene transcription with shRNA causes reduced SDH expression, and meantime, cell proliferation capacity is decreased. SDHA silencing combined with CDK inhibitors had a synergistic effect on cell cycle. Moreover, when using both SDHA inhibitors and CDK inhibitors, it can not only inhibit A2780 cell proliferation, but also inhibit cell invasion and metastasis ability. However, there was no difference between the total survival duration and progression-free survival periods, regardless of the enhanced TCA pathway or not (24).

## Conclusion

This article addresses SA metabolic abnormalities and the potential possible mechanisms of SA in ovarian cancer. Existing literature shows that metabolic reprogramming plays an important role in the occurrence of ovarian cancer, and that SA, as a signaling molecule, is a relatively important potential target to provide help in future diagnosis and treatment

## The Shortcomings

Part of the existing literature are conclusions obtained through data mining and further experimental verification is required. Moreover, the relevant experimental environment is set at 20%O_2_. This is also different from the tumor in the physical environment. And the high heterogeneity of ovarian cancer will also lead to the impact on the experimental results. Therefore, in the future, both *in vivo* and *in vitro* experiments will need more accurate research.

## Author Contributions

LX, HZ, and XW contributed equally to this work and should be considered co-first authors. All authors contributed to the article and approved the submitted version.

## Conflict of Interest

The authors declare that the research was conducted in the absence of any commercial or financial relationships that could be construed as a potential conflict of interest.

## Publisher’s Note

All claims expressed in this article are solely those of the authors and do not necessarily represent those of their affiliated organizations, or those of the publisher, the editors and the reviewers. Any product that may be evaluated in this article, or claim that may be made by its manufacturer, is not guaranteed or endorsed by the publisher.
